# Influence of the Vertical Emittance on the Calculability of the Synchrotron Ultraviolet Radiation Facility

**DOI:** 10.6028/jres.107.035

**Published:** 2002-10-01

**Authors:** U. Arp

**Affiliations:** National Institute of Standards and Technology, Gaithersburg, MD 20899-8410

**Keywords:** calculability, electron beam emittance, storage ring, synchrotron radiation, weak focusing

## Abstract

A method to include the influence of the vertical electron beam emittance onto the calculability of synchrotron radiation is introduced. It accounts for the finite vertical size and angular spread of the electron beam through a convolution procedure. The resulting angular spread of synchrotron radiation can differ significantly from the ideal Schwinger result, depending on the conditions. For the Synchrotron Ultraviolet Radiation Facility detailed results on the influence of the electron emittance for total power and polarization calculations are presented.

## 1. Introduction

**Fig. 1:**
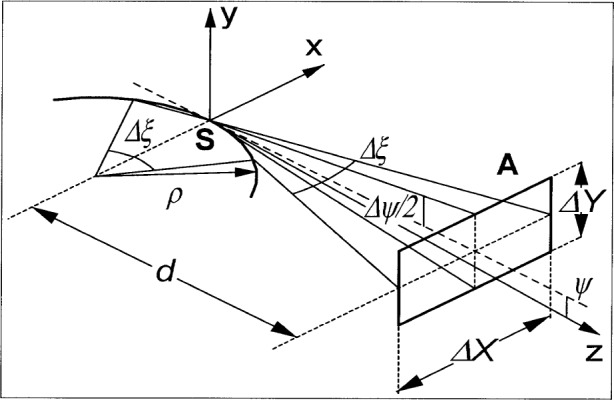
Geometry of the problem: An aperture A is positioned at distance *d* from the source point **S**. The aperture is assumed to be rectangular with horizontal (vertical) size **Δ***X*(**Δ***Y*).

The calibration of light sources using storage rings is based on the calculability of synchrotron radiation [1–4]. The ultimate uncertainty of these calibrations is determined by the accuracy with which all necessary parameters to calculate the radiation output can be determined. Fig. 1 illustrates the problem. The question is, how much radiation passes through the aperture A, which is positioned at distance *d* from the source point **S**. If the emittance, i.e., the phase space area occupied by the electron beam [5], of the electron beam and diffraction effects are neglected, this radiation output is a function of electron energy *E*, bending radius ***ρ***, wavelength ***λ***, and bandwidth **Δ*λ***, electron beam current *I*_B_, distance *d*, width **Δ***X* and height **Δ***Y* of the aperture, and the angle between the vertical center of the aperture and the orbital plane of the electrons ***ψ***.

At the Synchrotron Ultraviolet Radiation Facility SURF III [6] the electron energy and orbital radius are determined by the magnetic flux density *B* and the radio-frequency ***ν***_RF_. The Lorentz-force equation for the ideal orbit [7] gives the electron energy

$$E=\frac{B\;e\;c^{2}}{\pi \;\nu_{\text{RF}}},$$ (1)

and the synchronicity condition [7] delivers the orbital radius

$$\rho =\sqrt{{\left(\frac{c}{\pi \;\nu_{\text{RF}}}\right)}^{2}-{\left(\frac{m_{e}\;c}{B\;e}\right)}^{2}}.$$ (2)

*e* is the elementary charge, *m*_e_ the electron rest mass, and *c* the speed of light in vacuum. The electron beam current is determined using a well established electron counting procedure [8,9].

The determination of the geometrical factors is often quite challenging as well. One has to determine the distance *d* between the defining aperture A and source point **S** with high accuracy.

## 2. Vertical Electron Beam Parameters in Equilibrium

For a weak-focusing storage ring like SURF, most equilibrium parameters can be calculated analytically. The vertical emittance is given by [7]

$$\epsilon_{y}=\frac{C_{q}\;\beta_{y}}{J_{y}\;\rho },$$ (3)

where *C*_q_ is Sands’ quantum excitation constant [10],***β****_y_* the vertical betatron function, *J_y_* the vertical damping partition number, and ***ρ*** the orbital radius. For SURF, the vertical betatron function is determined by the orbital radius and the magnetic field index

$${n=\frac{\rho }{B}\frac{\partial B}{\partial r}|}_{r=\rho },$$ (4)

[11] through $\beta_{y}=\rho /\sqrt{n}=1.086$ m. The equilibrium vertical Gaussian beam size is given by [7]

$$\sigma_{y}=\sqrt{\epsilon_{y}\;\beta_{y}}$$ (5)

and the vertical Gaussian angular spread of the electron beam by [7]

$$\sigma_{y}^{\prime }=\sqrt{\frac{\epsilon_{y}}{\beta_{y}}}.$$ (6)

Assuming *E* = 380 MeV, ***ρ*** = 837.224 mm, and *n* = 0.594 the theoretical numbers for SURF are

$$\epsilon_{y}=\sigma_{y}^{\prime }\cdot \sigma_{y}=0.677\;\mu \text{rad}\cdot 0.732\;\mu m.$$

In reality the vertical emittance is larger than this theoretical value, because of coupling between the horizontal and vertical motion. From the measured vertical beam size [12] the coupling is estimated to be of order 1 %.

## 3. Excitation of the Vertical Betatron Motion

The bunch volume at SURF is very small in its natural state, causing unacceptably short lifetimes of the electron beam. To extend the lifetime the vertical betatron oscillation is excited [13,14], causing the vertical angular spread and beam size to increase. If the vertical beam size is measured, the new emittance can be calculated, because the vertical betatron function depends only on the magnetic lattice [5] and will remain unchanged as the beam size changes. The following calculation depends on the assumption that the beam profiles remain Gaussian in shape and the beam is stable [14,15]. The new emittance is

$$\epsilon_{y1}=\frac{\sigma_{y1}^{2}}{\beta_{y}}$$ (7)

and the new vertical angular spread of the electron beam

$$\sigma_{y1}^{\prime }=\sqrt{\frac{\epsilon_{y1}}{\beta_{y}}}=\frac{\sigma_{y1}}{\beta_{y}}.$$ (8)

For example, if the vertical beam size is enlarged to ***σ****_y_*_1_ = 0.425 mm, which corresponds to a vertical full-width-at-half-maximum (FWHM) of 1 mm, the values are ***ϵ****_y_*_1_ = 1.660 × 10^5^ **μ**m **μ**rad and ${\sigma^{\prime }}_{y1}=391$ **μ**rad.

## 4. Vertical Angular Distribution of Synchrotron Radiation

The vertical angular distribution of the emitted synchrotron radiation can be calculated using Schwinger’s equation [16]. We can separate the contributions into radiation with electrical vector parallel *P*_║_(***ψ, λ***) and perpendicular *P*_⊥_(***ψ, λ***) to the orbital plane of the electrons. ***λ*** is the wavelength of the emitted radiation and ***ψ*** is the angle relative to the orbital plane of the electrons (see Fig. 1). The total power is *P*_tot_(***ψ, λ***) = *P*_║_(***ψ, λ***) + *P*_⊥_(***ψ, λ***).

$$\begin{array}{r} P_{\|}\left(\psi ,\;\lambda \right)=\frac{2}{3\;\epsilon_{0}}\frac{e\;\rho^{2}\;\Delta \xi }{\gamma^{4}\;\lambda^{4}}\frac{I_{B}}{\beta }\\ \Delta \lambda {\left[1+{\left(\gamma \psi \right)}^{2}\right]}^{2}K_{2/3}{\left[\zeta \left(\lambda ,\;\psi \right)\right]}^{2}\\ P_{⊥}\left(\psi ,\;\lambda \right)=\frac{2}{3\;\epsilon_{0}}\frac{e\;\rho^{2}\;\Delta \xi }{\gamma^{4}\;\lambda^{4}}\frac{I_{B}}{\beta } \end{array}$$ (9)

$$\Delta \lambda \left[1+{\left(\gamma \psi \right)}^{2}\right]{\left(\gamma \psi \right)}^{2}K_{1/3}{\left[\zeta \left(\lambda ,\;\psi \right)\right]}^{2}.$$ (10)

This formulation is only applicable to SURF and its unique geometry. *e* is the elementary charge, **Δ*ξ*** the horizontal acceptance angle, ***ρ*** the orbital radius, relativistic ***γ*** = *E*/(*m*_e_ *c*^2^) and $\beta =\sqrt{1-\gamma^{-2}}$, electron beam current *I*_B_, and bandwidth **Δ*λ***. *K*_2/3_[ζ(***λ, ψ***)] and *K*_1/3_[ζ(***λ, ψ***)] are modified Bessel-functions of fractional order and $\zeta \left(\lambda ,\;\psi \right)=\frac{\lambda_{c}}{2\lambda }{\left[1+{\left(\gamma \psi \right)}^{2}\right]}^{3/2}$ with the characteristic wavelength ***λ***_c_ = 4**π *ρ***/(3***γ***^3^).

## 5. Influence of the Emittance on the Vertical Angular Spread of Synchrotron Radiation

Schwinger’s equation [16] is useful to calculate the synchrotron radiation emission of one electron in a perfect orbit, but does not take into account the electron emittance. This calculated vertical angular distribution of the synchrotron radiation has to be convolved with the vertical angular spread of the electron beam and also the angular spread caused by the finite vertical beam size, which depends on the distance from the tangent point.

To simplify the procedure one can convolve the vertical angular spread of the electron beam with the contribution from the beam size first. Both are assumed to be Gaussian and the convolution of two Gaussians with widths ***σ***_1_ and ***σ***_2_ results in a Gaussian with total width $\sigma_{\text{tot}}=\sqrt{\sigma_{1}^{2}+\sigma_{2}^{2}}$. For the total angular spread caused by the emittance of the electron beam this leads to

$$\sigma_{y\text{tot}}^{\prime }=\sqrt{{\sigma_{y1}^{\prime }}^{2}+{\left(\frac{\sigma_{y1}}{d}\right)}^{2}},$$ (11)

where *d* is the distance from the point of observation to the tangent point.

Next, the total vertical angular spread of the emitted synchrotron radiation is the convolution of *P*_tot_(***ψ, λ***) with a Gaussian of width ${\sigma^{\prime }}_{y\text{tot}}$. The convolution integral is [17]

$$\begin{array}{c} P_{\text{tot}}^{1}\left(\psi ,\;\lambda \right)=\int_{-\infty }^{+\infty }P_{\text{tot}}\left(\psi -y,\;\lambda \right)\cdot \text{exp}\left(\frac{-y^{2}}{2{\sigma_{y\text{tot}}^{\prime }}^{2}}\right)dy\\ =P_{\text{tot}}\left(\psi ,\;\lambda \right)*\text{exp}\left(\frac{-\psi^{2}}{2{\sigma_{y\text{tot}}^{\prime }}^{2}}\right). \end{array}$$ (12)

This convolution integral has to be solved numerically. By applying the convolution theorem one can solve Eq. (12) easily. If *X* = *FT*(*x*) denotes the Fourier-transform of function *x* and *x* = *IFT*(*X*) its inverse Fourier-transform, the convolution can be written as

$$P_{\text{tot}}^{1}\left(\psi ,\;\lambda \right)=IFT\left(FT\left[P_{\text{tot}}\left(\psi ,\;\lambda \right)\right]\cdot FT\left[\text{exp}\left(\frac{-\psi^{2}}{2{\sigma_{y\text{tot}}^{\prime }}^{2}}\right)\right]\right).$$ (13)

**Fig. 2:**
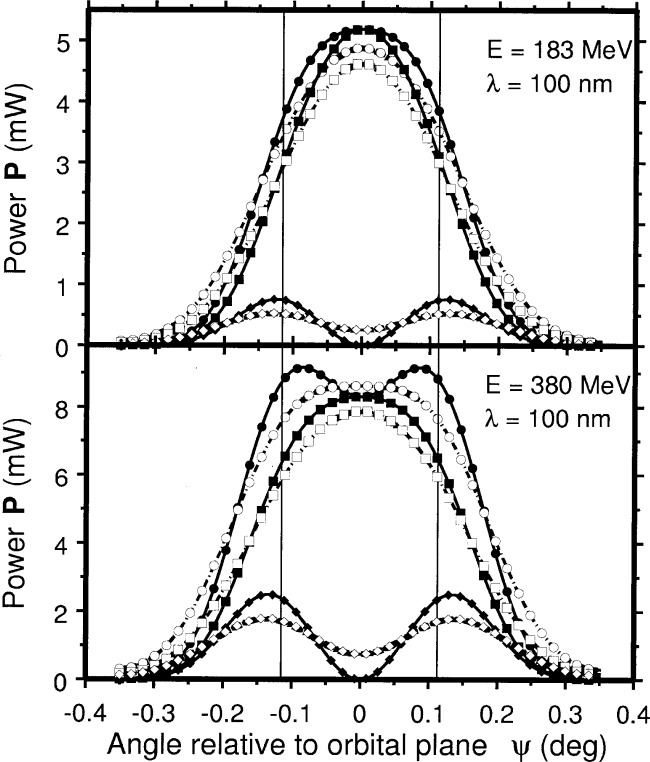
Ideal vertical distribution of the synchrotron radiation at ***λ*** = 100 nm, *I*_B_ = 100 mA, **Δ*λ*** = 0.01 ***λ***, *P*_tot_(***ψ, λ***) (●), *P*_║_(***ψ, λ***) (■), and *P*_⊥_(***ψ, λ***) (◆), as well as the calculated distribution taking into account emittance effects $P_{\text{tot}}^{1}\left(\psi ,\;\lambda \right)\;\left(○\right)$, $P_{\|}^{1}\left(\psi ,\;\lambda \right)\;\left(□\right)$, and $P_{⊥}^{1}\left(\psi ,\;\lambda \right)\;\left(⋄\right)$. The calculation was performed for a vertical Gaussian beam size ***σ****y*_1_ = 1 mm at distance *d* = 2500 mm. Top: *E* = 183 MeV, ***ρ*** = 837.2217 mm, Bottom: *E* = 380 MeV, ***ρ*** = 837.2242 mm. The vertical lines denote the integration limits for the later total flux and polarization calculations.

In Fig. 2 results are shown for SURF for *d* = 2500 mm, ***σ****_y_*_1_ = 1 mm, and ***λ*** = 100 nm, for both *E* = 380 MeV and *E* = 183 MeV.

## 6. Optical Power Passing Through an Aperture

To calculate the power passing through the aperture A of vertical size **Δ***Y* and horizontal size **Δ***X*, positioned at distance *d* from the source point **S**, the result of the convolution in Eq. (12) has to be integrated over the vertical angle. The integration over the horizontal acceptance angle is trivial, since the horizontal distribution is flat, and can be replaced by a multiplication [factor **Δ*ξ*** = **Δ***X*/*d* in Eqs. (9) and (10)]. To keep things simple we assume ***ψ*** = 0 (vertical center of the aperture is in plane with the electron orbit).

$$P_{\text{tot}}^{A^{1}}\left(\lambda \right)=\int_{-\Delta \psi /2}^{+\Delta \psi /2}P_{\text{tot}}^{1}\left(\psi ,\;\lambda \right)d\psi$$ (14)

**Fig. 3:**
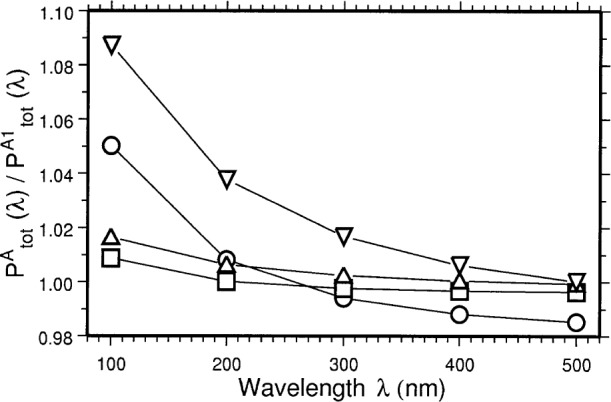
Ideal optical power $P_{\text{tot}}^{A}\left(\lambda \right)$ divided by the optical power including emittance effects $P_{\text{tot}}^{A^{1}}\left(\lambda \right)$ for *d* = 2500 mm, **Δ***X* = **Δ***Y* = 10 mm. *E* = 380 MeV, ***σ****_y_*_1_ = 1 mm (○), *E* = 380 MeV, ***σ****_y_*_1_ = 0.425 mm (□), *E* = 183 MeV, ***σ****_y_*_1_ = 1 mm (∇), and *E* = 183 MeV, ***σ****_y_*_1_ = 0.425 mm (**Δ**).

Since the result of Eq. (13) was produced numerically, the integration in Eq. (14) has to be performed numerically as well. In Fig. 3 results are shown for different electron energies and vertical beam sizes. In this example for a 1 mm vertical beam size at 183 MeV and ***λ*** = 100 nm, the change relative to the ideal Schwinger value of the optical power is about 9 %.

## 7. Polarization of Radiation Passing Through an Aperture

The degree of linear polarization for synchrotron radiation is defined as [18]

$$D_{\text{lin}}\left(\psi ,\;\lambda \right)=\frac{P_{\|}\left(\psi ,\;\lambda \right)-P_{⊥}\left(\psi ,\;\lambda \right)}{P_{\|}\left(\psi ,\;\lambda \right)+P_{⊥}\left(\psi ,\;\lambda \right)}.$$ (15)

If the degree of linear polarization of all the radiation passing through the aperture A is searched, Eq. (15) has to be integrated

$$D_{\text{lin}}^{A}\left(\lambda \right)=\int_{\frac{-\Delta \psi }{2}}^{\frac{+\Delta \psi }{2}}\frac{P_{\|}\left(\psi ,\;\lambda \right)-P_{⊥}\left(\psi ,\;\lambda \right)}{P_{\|}\left(\psi ,\;\lambda \right)+P_{⊥}\left(\psi ,\;\lambda \right)}d\psi .$$ (16)

To account for the emittance the two polarization contributions have to be convoluted the same way as the total, and then numerically integrated over the vertical acceptance angle

$$D_{\text{lin}}^{A^{1}}\left(\lambda \right)=\int_{\frac{-\Delta \psi }{2}}^{\frac{+\Delta \psi }{2}}\frac{P_{\|}^{1}\left(\psi ,\;\lambda \right)-P_{⊥}^{1}\left(\psi ,\;\lambda \right)}{P_{\|}^{1}\left(\psi ,\;\lambda \right)+P_{⊥}^{1}\left(\psi ,\;\lambda \right)}d\psi .$$ (17)

**Fig. 4:**
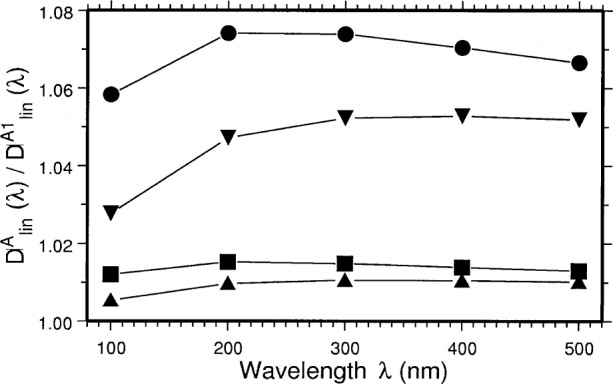
Ideal degree of linear polarization of the radiation passing through the aperture A $D_{\text{lin}}^{A}\left(\lambda \right)$ divided by the degree of linear polarization including the vertical emittance $D_{\text{lin}}^{A^{1}}\left(\lambda \right)$ for *d* = 2500 mm, **Δ***X* = **Δ***Y* = 10 mm. *E* = 380 MeV, ***σ****_y_*_1_ = 1 mm (●), *E* = 380 MeV, ***σ****_y_*_1_ = 0.425 mm (■), *E* = 183 MeV, ***σ****_y_*_1_ = 1 mm (▼), and *E* = 183 MeV, ***σ****_y_*_1_ = 0.425 mm (▲).

Again Fig. 4 shows clearly large deviations from the ideal Schwinger values for the polarization.

## 8. Conclusions

The influence of the vertical electron beam emittance on the vertical distribution of synchrotron radiation has been analyzed for SURF III.

For the total power passing through an aperture and the polarization of the radiation it was found that deviations from the ideal Schwinger values can be of order several percent, depending on the actual conditions. The deviations are most prominent if the vertical acceptance angle **Δ*ψ*** is of the same order as the FWHM of the vertical angular distribution, as illustrated in Fig. 2. If all radiation is accepted vertically there is no difference to expect. If the vertical distribution is much wider than the vertical acceptance, the differences are expected to be small.

However, it is important to point out that this model only works well when the shape of the beam is Gaussian and no instabilities are present.

## References

[1] Kostkowski HJ, Lean JL, Saunders RD, Hughey LR (1986). Appl Opt.

[2] Thornagel R, Fischer J, Friedrich R, Stock M, Ulm G, Wende B (1996). Metrologia.

[3] Zama T, Saito T, Onuki H (1998). J Synchr Rad.

[4] Zama T, Saito T, Onuki H (1999). J Electron Spectrosc Relat Phenom.

[5] Chao AW, Tigner M (1999). Handbook of Accelerator Physics and Engineering.

[6] Furst ML, Graves RM, Hamilton A, Hughey LR, Madden RP, Vest RE, Trzeciak WS, Bosch RA, Greenler L, Wahl PRD, Luccio A, MacKay W (1999).

[7] Wiedemann H (1993). Particle Accelerator Physics.

[8] Hughey LR, Schaefer AR (1982). Nucl Inst Meth Phys Res A.

[9] Schaefer AR, Hughey LR, Fowler JB (1984). Metrologia.

[10] Sands M (1971). Physics with Intersecting Storage Rings.

[11] Arp U, Friedman R, Furst ML, Makar S, Shaw P-S (2000). Metrologia.

[12] Arp U (2001). Nucl Inst Meth Phys Res A.

[13] Rakowsky G, Hughey LR (1979). IEEE Trans Nucl Sci.

[14] Arp U, Lucatorto TB, Harkay K, Kim K-J (2002). Rev Sci Inst.

[15] Arp U, Fraser GT, Walker AR Hight, Lucatorto TB, Lehmann KK, Harkay K, Sereno N, Kim K-J (2001). Phys Rev ST Accel Beams.

[16] Schwinger J (1949). Phys Rev.

[17] Press WH, Teukolsky SA, Vetterling WT, Flannery BP (1992). Numerical Recipes in C.

[18] Duke PJ (2000). Synchrotron Radiation, Vol. 3 of Oxford Series on Synchrotron Radiation.

